# Hydroxyl radicals dominate reoxidation of oxide-derived Cu in electrochemical CO_2_ reduction

**DOI:** 10.1038/s41467-022-31498-8

**Published:** 2022-06-27

**Authors:** Shijia Mu, Honglei Lu, Qianbao Wu, Lei Li, Ruijuan Zhao, Chang Long, Chunhua Cui

**Affiliations:** Molecular Electrochemistry Laboratory, Institute of Fundamental and Frontier Sciences, University of Electronic Science and Technology of China, Chengdu, 610054 China

**Keywords:** Electrocatalysis, Heterogeneous catalysis, Catalytic mechanisms, Electrocatalysis

## Abstract

Cu^δ+^ sites on the surface of oxide-derived copper (OD-Cu) are of vital importance in electrochemical CO_2_ reduction reaction (CO_2_RR). However, the underlying reason for the dynamically existing Cu^δ+^ species, although thermodynamically unstable under reductive CO_2_RR conditions, remains uncovered. Here, by using electron paramagnetic resonance, we identify the highly oxidative hydroxyl radicals (OH^•^) formed at room temperature in HCO_3_^-^ solutions. In combination with in situ Raman spectroscopy, secondary ion mass spectrometry, and isotope-labelling, we demonstrate a dynamic reduction/reoxidation behavior at the surface of OD-Cu and reveal that the fast oxygen exchange between HCO_3_^-^ and H_2_O provides oxygen sources for the formation of OH^•^ radicals. In addition, their continuous generations can cause spontaneous oxidation of Cu electrodes and produce surface CuO_x_ species. Significantly, this work suggests that there is a “seesaw-effect” between the cathodic reduction and the OH^•^-induced reoxidation, determining the chemical state and content of Cu^δ+^ species in CO_2_RR. This insight is supposed to thrust an understanding of the crucial role of electrolytes in CO_2_RR.

## Abstract (web summary)

The Cu^δ+^ species has been frequently detected during CO_2_ reduction and plays a key role in C-C coupling for C2+ products. Here the authors uncover that the HCO_3_^-^ solution can activate hydroxyl radicals that oxidize metallic Cu to surface Cuδ+ species.

## Introduction

Conversion of CO_2_ into value-added chemicals through renewable electricity-powered electrochemical CO_2_ reduction reaction (CO_2_RR) has been recognized as a promising strategy to achieve “carbon-neutral”^1–3^. Oxide-derived copper (OD-Cu) has been proven as a group of efficient electrocatalysts for CO_2_RR, especially for multi-carbon products (C_2+_)^4–6^. The precise mechanism remains unknown and different views have been proposed^7–14^. Specifically, both experiments and theoretical calculations demonstrated that the Cu^δ+^/Cu^0^ interface can activate the inert CO_2_ molecules and promote CO-CO coupling^10,11^. To regulate the selectivity of C_2+_ products, many efficient OD-Cu catalysts with characteristic Cu^δ+^ sites have been achieved through constructing various oxidized pre-catalysts or employing CO_2_-pulsed electrolysis^7–9,12^.

Thermodynamically, CuO_x_ phases should be removed under the CO_2_RR conditions thereby the loss of the active Cu^δ+^ species^15^. While some studies have demonstrated the reduction of CuO_x_ phases to metallic Cu during CO_2_RR^16–20^. Interestingly, despite these, the Cu^δ+^ species has been frequently detected in CO_2_RR^8,9,21–23^. This puzzling phenomenon leads to divergent views of the presence of Cu^δ+^ species. Cuenya et al. demonstrated that the O_2_ plasma-treated CuO_x_ is resistant to reduction^24^. Yu et al. found that in situ generated CO_2_RR intermediates on the surface of OD-Cu stabilize Cu^δ+^ species^8^. Chen et al. pointed out that the chemical states of Cu are associated with the oxidation caused by an uncertain oxidative species in the electrolytes^25–27^. We took the view that the Cu^δ+^ species should be dynamically existing, and we propose that the redox conditions provided by electrochemical cathodic reduction and oxidative species in electrolytes should be crucial. Understanding the reduction/reoxidation behavior of OD-Cu in commonly used CO_2_-saturated KHCO_3_ electrolytes is a grand challenge but vital to identifying what is the oxidative species.

Here, using in situ Raman spectroscopy, we observe the rapid reoxidation phenomenon of Cu to Cu_2_O species within a very short time scale (10 s) upon stopping the cathodic potential, and we record the dynamically existing Cu^δ+^ species at the surface of OD-Cu during the CO_2_RR. We further identify that the rapid reoxidation is caused by strongly oxidative OH^•^ radicals existing in KHCO_3_ solutions, by using electron paramagnetic resonance (EPR). With the isotope-labeling technique, we point out that the OH^•^ radicals are generated from both HCO_3_^−^ and H_2_O upon oxygen exchange in HCO_3_^−^ aqueous solutions at room temperature. In addition, owing to the continuous generation of OH^•^ radicals, we observe higher degrees of oxidizing corrosion of Cu electrodes in CO_2_- or Ar-saturated KHCO_3_ solution under open circuit potential (OCP) relative to those electrolytes without OH^•^ radicals, giving a hint of oxidative KHCO_3_ electrolytes. This work demonstrates unexpected OH^•^ radicals as the oxidative species, and it guides the fundamental understanding of the origin of Cu^δ+^ species in CO_2_RR.

## Results

### Dynamic reduction/reoxidation behavior

To enhance in situ Raman signals, a surface roughened OD-Cu electrode was selected as a model catalyst for this study^4,28^. It was prepared via depositing the micro-nano Cu particles onto the surface of the Cu mesh substrate (Supplementary Fig. 1), by using a modified electrodeposition method^29^. The as-prepared Cu electrodes show a mainly metallic Cu state, with surfaces being oxidized to Cu_2_O phases owing to the exposure to air after electrodeposition and KHCO_3_ electrolytes before applying potentials for CO_2_RR (Supplementary Fig. 1).

**Fig. 1:**
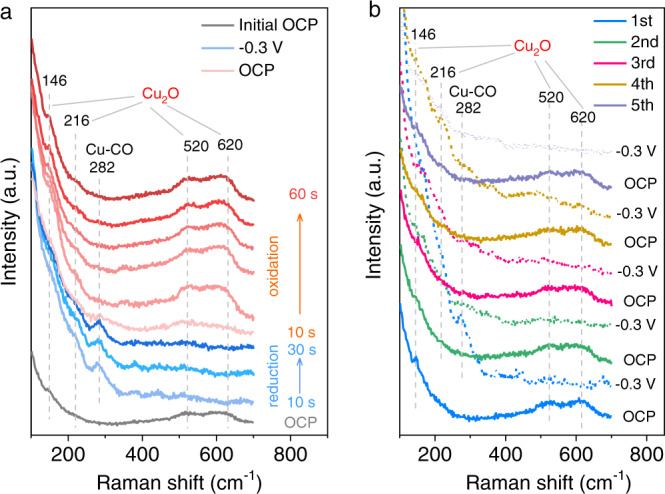
In situ Raman spectra of OD-Cu electrodes in CO_2_-saturated 0.5 M KHCO_3_. **a** Real-time Raman spectra of surface Cu_2_O species at −0.3 V_RHE_ and subsequently at open circuit potential (OCP). **b** Raman spectra under loop tests with a reduction potential at −0.3 V_RHE_ for 10 s and reoxidation potential at OCP for 20 s.

We implemented potential-dependent Raman spectra to investigate the stability of surface Cu_2_O species under cathodic potentials in CO_2_-saturated 0.5 M KHCO_3_ solution (Supplementary Fig. 2). The vibration bands of surface Cu_2_O were observed at ~216, ~520, and ~620 cm^−1^ at >0.0 V versus reversible hydrogen electrode (V_RHE_)^28,30,31^. While, at <−0.2 V_RHE_, the typical Cu_2_O signals disappear, indicating the surface Cu^+^ species was reduced to metallic Cu. This result is supported by the prediction of the Pourbaix diagram for Cu and the previous reports^15–20^. In addition, the peaks at 282, 360, 2070–2100 cm^−1^ are related to the frustrated *ρ*(Cu–C–O) rotational mode, *ν*(Cu–CO) stretching mode, and intramolecular C≡O stretching vibration of CO intermediates, respectively. The bands at 2820–2950 cm^−1^ are assigned to the -CH_x_ stretching regions^30,31^ from 0.2 to −0.6 V_RHE_ (Fig. 1a, b and Supplementary Figs. 2–4), demonstrating the initiation of CO_2_RR. Thus, to reduce surface Cu_2_O to metallic Cu (Supplementary Figs. 2 and 5) and to avoid the reconstruction of Cu at very negative potentials during CO_2_RR^18,32^, a moderate reduction potential of −0.3 V_RHE_ was chosen for further Raman study.

The reoxidation of OD-Cu surface was investigated via real-time Raman test, by applying a reduction potential at −0.3 V_RHE_ for 30 s firstly, and then switching to OCP for 60 s. The spectra were acquired every 10 s. As shown in Fig. 1a, once switching the potential from −0.3 V_RHE_ to OCP, the metallic Cu was rapidly oxidized to Cu_2_O species within 10 s, and its three characteristic bands at 146, ~520, and ~620 cm^−1^ re-appeared after 20 s^28,30,31^. This rapid reoxidation phenomenon indicates a strongly oxidative species existing in the KHCO_3_ electrolyte, in contrast to the non-reoxidation process of OD-Cu in the Ar-saturated 0.25 M K_2_SO_4_ electrolyte (Supplementary Fig. 6). Here K_2_SO_4_ was selected as a control electrolyte because of its moderate solubility relative to KClO_4_ (~0.12 M), suitable chemical stability, and relatively weaker interaction with Cu in contrast to such as KCl and KI.

To further confirm the reduction/reoxidation phenomenon, we implemented a loop test where alternate potentials between OCP for 20 s and −0.3 V_RHE_ for 10 s were employed. As shown in Fig. 1b, during the five cycles, we observed that the Cu_2_O phase disappears at −0.3 V_RHE_ and re-produces at OCP. We found that the surface Cu species go through the process: Cu_2_O → CuO_x_ → metallic Cu, with the cathode potential decreasing from OCP to −0.3 V_RHE_ (Supplementary Fig. 2). It is a reverse process when switching from −0.3 V_RHE_ to OCP. Thus, we suggest that the chemical state of Cu and the phase of surface Cu species are the results of dynamic equilibrium between the cathodic reduction and the reoxidation caused by strongly oxidative species in KHCO_3_ electrolytes. This explanation may reconcile the debates on the stability of Cu^δ+^ species during CO_2_RR observed from different groups^8,9,16–23^.

To preclude the effect of trace undesired oxidative species, such as residual O_2_, in the electrolyte or at the electrode surface, we implemented a long-time reduction of electrodeposited Cu electrode in CO_2_-saturated KHCO_3_ solution at −0.3 V_RHE_ for 20 min. Then we switched the potential to OCP while recording the time-dependent optical microscopic images of the electrode surface (Supplementary Fig. 7). Within 10 min, the bright Cu surface gradually converted into a light-black surface, indicating an explicit oxidation process of Cu in the bulk KHCO_3_ electrolyte.

### OH^•^ radicals in KHCO_3_ electrolytes

Inspired by the formation of various radicals in the KHCO_3_ system^33–37^, we speculate that there may be free radicals in the as-used KHCO_3_ electrolytes. Thus, EPR spectroscopy was applied to investigate the radical species in a 0.5 M KHCO_3_ aqueous solution. Considering the very short lifetimes of radicals (up to several ms), 5,5-dimethyl-1-pyrroline N-oxide (DMPO) as a spin trapping agent was added into KHCO_3_ electrolytes, thus the formed DMPO-radical adducts have the lifetimes as long as minute-scale^38^, facilitating the EPR tests.

**Fig. 2:**
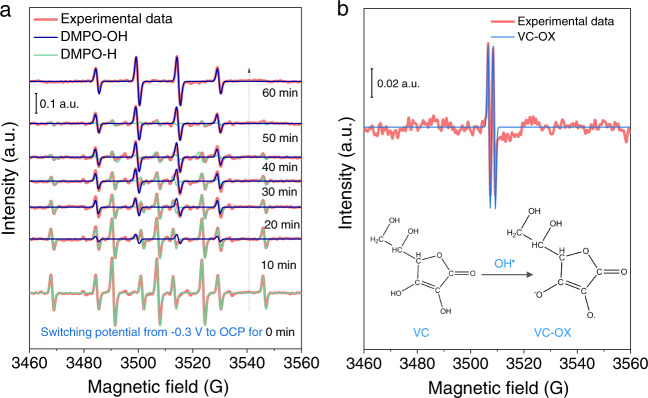
EPR spectra were recorded in a CO_2_-saturated 0.5 M KHCO_3_ electrolyte containing 100 mM DMPO. **a** Time-dependent EPR spectra of the KHCO_3_ solution after switching potential from −0.3 V_RHE_ (20 min reduction) to OCP for different periods. **b** EPR spectra of the KHCO_3_ solution with 10 mM VC. Inset shows the structure transition from VC and oxidized VC-OX caused by OH^•^. The simulated EPR spectra of DMPO-OH, DMPO-H, and VC-OX adducts were obtained according to their hyperfine splitting constants.

To eliminate any pre-introduced oxidative species, a 20 min of long-time reduction on the Cu electrode at −0.3 V_RHE_ was performed in CO_2_-saturated 0.5 M KHCO_3_ containing 100 mM DMPO. At −0.3 V_RHE_, the hydrogen evolution reaction (HER) occurs and the generated hydrogen radicals (H^•^) can be trapped as a DMPO-H adduct (hyperfine splitting constants, A_N_ = 1.65 mT, A_H_ = 2.25 mT)^38^. Once the potential was switched to OCP, immediately the radicals in the solution were tested through a real-time EPR. As shown in Fig. 2a, DMPO-H radical adduct generated during the HER did not disappear as soon as we stopped the bias, owing to the increased lifetime, thus they can be measured even if we switched the potential from −0.3 V_RHE_ to OCP. Interestingly, after 10 min, the DMPO-OH (A_N_ = 1.50 mT, A_H_ = 1.48 mT)^39^ emerged and gradually became the dominant signal with increasing the time within 60 min. Based on this carefully designed test protocol, we claim that the newly generated OH^•^ radicals were indeed activated in the HCO_3_^−^ electrolyte. The continuously ascending OH^•^ radical intensity was further confirmed when resting the KHCO_3_ solution at OCP for 24 h without pre-reduction at −0.3 V_RHE_ (Supplementary Fig. 8).

We assume that the generation of fresh H^•^ radical will end once stopping the potential at −0.3 V_RHE_, thus it is reasonable that the DMPO-H signal decays owing to its limited lifetime (Fig. 2a). In contrast, the signal of DMPO-OH increased over 24 h (Supplementary Fig. 8), suggesting a continuous production of OH^•^ radicals with a considerable amount excited by the electrolyte, considering its half-time of DMPO-OH adduct (minute-scale)^40,41^. Besides, the Raman vibration of Cu-OH mode at 710 cm^−1^, which could be caused by OH^•^ radical, was observed during CO_2_RR from 0.2 to −0.3 V_RHE_ (Supplementary Fig. 2)^28,42^. OH^•^ radical is a strongly oxidative species, with a high electrode potential of 2.73 V versus normal hydrogen electrode^43^, which can lead to the rapid reoxidation of surface OD-Cu in KHCO_3_ electrolytes during CO_2_RR.

The control experiments showed that no relevant DMPO-OH signals were detected in both pure water and 0.25 M K_2_SO_4_ solutions containing 100 mM DMPO (Supplementary Fig. 9). Meanwhile, the reoxidation behavior of surface OD-Cu in 0.25 M K_2_SO_4_ electrolytes was not observed via the in situ Raman test (Supplementary Fig. 6). These results imply that pure water or K^+^ cations alone cannot produce OH^•^ radicals at room temperature. Nevertheless, we found that HCO_3_^−^ anions play a key role in the generation of OH^•^ radicals, by tuning the HCO_3_^−^/SO_4_^2−^ mole ratio under the same K^+^ concentration (0.5 M), where the DMPO-OH signals decreased with decreasing the HCO_3_^−^ concentrations (Supplementary Fig. 10).

To further verify the OH^•^ radicals, we added 10 mM vitamin C (VC) as an OH^•^ scavenger into the CO_2_-saturated 0.5 M KHCO_3_ solution containing 100 mM DMPO. As shown in Fig. 2b, the EPR signal of the DMPO-OH adduct disappeared and was replaced by a newly formed VC-OX radical (A_H_ = 0.18 mT) that is from VC oxidation (VC-OX) by OH^•^ radicals^44^. The inset in Fig. 2b displays the oxidation process. The oxidation phenomenon was further demonstrated via the color variation of VC KHCO_3_ solution from colorless to light yellow after 24 h aging (Supplementary Fig. 11). A control experiment excludes the VC-OX radicals from self-oxidation, where no EPR signals were observed in the 10 mM VC water solution containing 100 mM DMPO (Supplementary Fig. 12). Besides, the reoxidation of OD-Cu electrode at OCP was not observed after the reduction of surface Cu_2_O species to metallic Cu at −0.3 V_RHE_ in the CO_2_-saturated KHCO_3_ solution containing VC, by using in situ Raman spectroscopy (Supplementary Fig. 13).

**Fig. 3:**
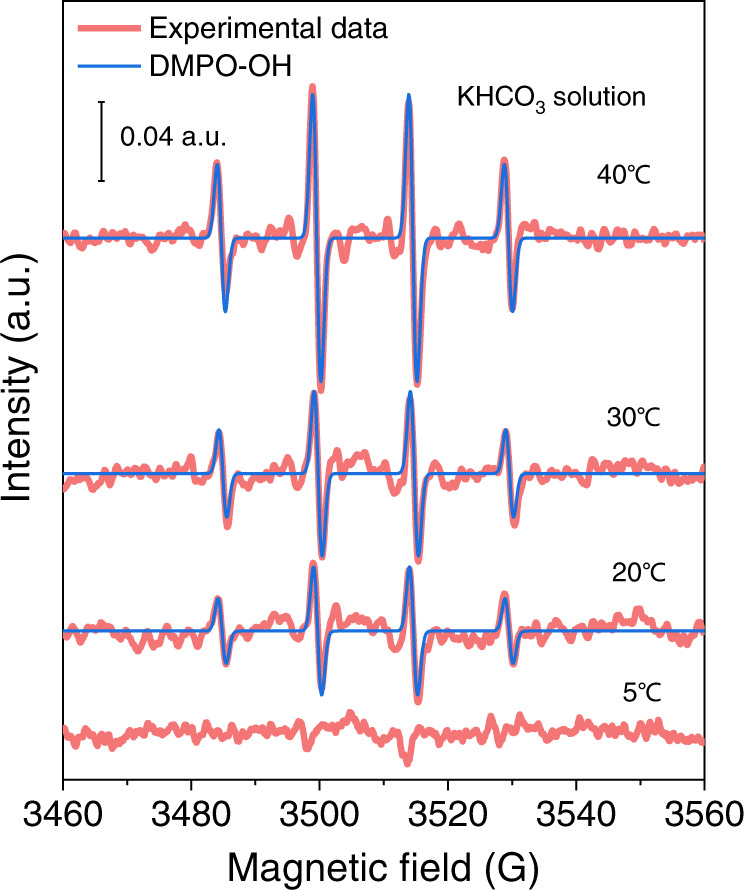
Temperature-dependent EPR spectra from 5 to 40 °C. EPR data were recorded in Ar-saturated 0.5 M KHCO_3_ solutions containing 100 mM DMPO without pre-reduction operations. Each spectrum was acquired after 8 h resting at the indicated temperature.

To consider whether the formation of OH^•^ radicals could be a thermally activated process, the signal of the DMPO-OH adduct has been tracked via a temperature-dependent study. We hypothesize that the room temperature may activate HCO_3_^−^ solutions to produce OH^•^ radicals. To confirm it, we implemented the temperature-dependent EPR measurements from 5 to 40 °C in an Ar-saturated 0.5 M KHCO_3_ electrolyte containing 100 mM DMPO (Fig. 3). When the temperature is as low as 5 °C, no EPR signals were detected. An obvious EPR signal from DMPO-OH was observed around 20 °C and increased with the enhanced temperature. This result indicates that temperature is a key parameter for OH^•^ radical generation in the KHCO_3_ solutions.

### Oxidizing corrosion of Cu plate in KHCO_3_ solution

**Fig. 4:**
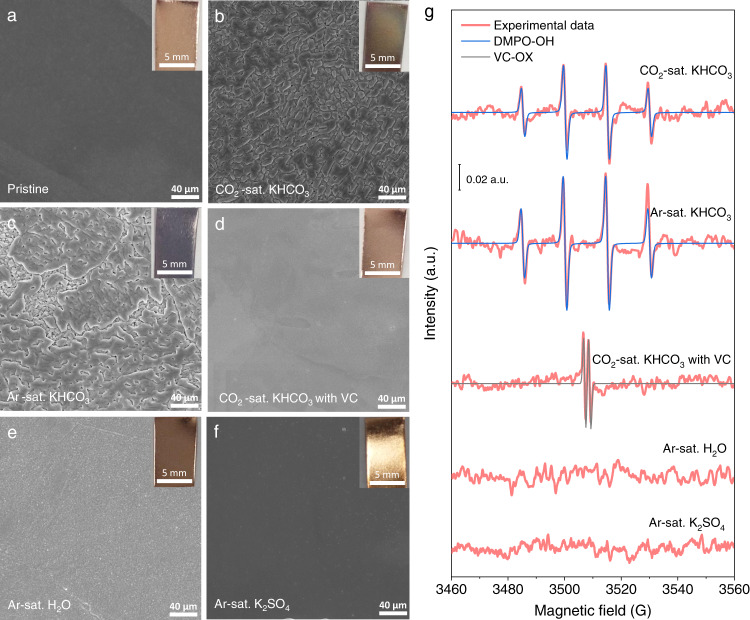
Oxidizing corrosion of the polished Cu plates in different solutions. SEM images of Cu plates before **a**, and after 24 h oxidizing corrosions in **b** CO_2_-saturated KHCO_3_, **c** Ar-saturated KHCO_3_, **d** CO_2_-saturated KHCO_3_ containing VC, **e** Ar-saturated ultrapure water, and **f** Ar-saturated K_2_SO_4_ solutions. Correspondingly, the photographs are displayed in insets. **g** EPR spectra of the corresponding solutions containing 100 mM DMPO after 24 h resting. The concentrations of KHCO_3_, K_2_SO_4,_ and VC are 0.5 M, 0.25 M, and 10 mM, respectively.

Given the steady generation of OH^•^ radicals in KHCO_3_ electrolytes, we supposed that it would result in a higher degree of oxidizing corrosion of Cu metal. A polished Cu plate (inset in Fig. 4a) was placed into a CO_2_-saturated KHCO_3_ solution. After 24 h resting, a visually light-black Cu surface was observed (inset in Fig. 4b). To preclude the CO_2_(aq) effect, the same operation was implemented in Ar-saturated KHCO_3_, and a darker Cu surface was observed associated with a stronger DMPO-OH signal (inset in Fig. 4c). Thus, the Cu oxidation by CO_2_(aq) was ruled out. To further ascertain Cu oxidation by electrolyte-induced OH^•^, the control experiments were carried out in KHCO_3_ solution containing VC, pure water, and K_2_SO_4_ solution, respectively. No obvious color changes were visually seen for the three cases (insets in Fig. 4d–f), compared to the pristine Cu plate (inset in Fig. 4a).

The surface morphology of polished Cu plates before and after oxidizing corrosions was characterized by using an optical microscope (Supplementary Fig. 14) and scanning electron microscopy (SEM, Fig. 4a–f). The oxidized surfaces were observed in CO_2_- and Ar-saturated KHCO_3_ solutions (Fig. 4b, c), relative to pristine Cu (Fig. 4a), KHCO_3_ solution with VC (Fig. 4d), pure water (Fig. 4e), and K_2_SO_4_ solution (Fig. 4f). Moreover, SEM images showed that the thicknesses of oxide layers at Cu plates reach ~596 and ~780 nm in CO_2_- and Ar-saturated KHCO_3_ solutions, respectively (Supplementary Fig. 15). The phase analyses by X-ray diffraction (Supplementary Fig. 16) and high-resolution transmission electron microscopy (HRTEM, Supplementary Fig. 17) illustrated that the corrosion products mainly consist of Cu_2_O. After corrosions, the atomic content of the O element determined by energy-dispersive spectroscopy (Supplementary Figs. 18–23) increased from pristine 0.65% to 14.72% and 19.82% in CO_2_- and Ar-saturated KHCO_3_ solutions respectively, while no obvious variations were discerned for the other three cases (Supplementary Fig. 24).

The oxidizing corrosion degrees are well related to the amount of OH^•^ radicals. As shown in Fig. 4g, after 24 h resting, the CO_2_- and Ar-saturated KHCO_3_ solutions display obvious EPR signals of DMPO-OH and the latter has a higher intensity. By comparison, there was no signal for the other three solutions, except for the KHCO_3_ solution with VC that presents an EPR signal of VC-OX due to VC oxidation by OH^•^ radicals. These results are further supported by analyzing the surface chemical states of Cu 2*p* of oxidized Cu plates using X-ray photoelectron spectroscopy (XPS), where a little Cu^2+^ species with deep oxidation was found for CO_2_- and Ar-saturated KHCO_3_ solutions, while only Cu^1+^/Cu^0^ species were identified for the other cases (Supplementary Fig. 25).

### Sources of OH^•^ radicals in KHCO_3_ solutions

**Fig. 5:**
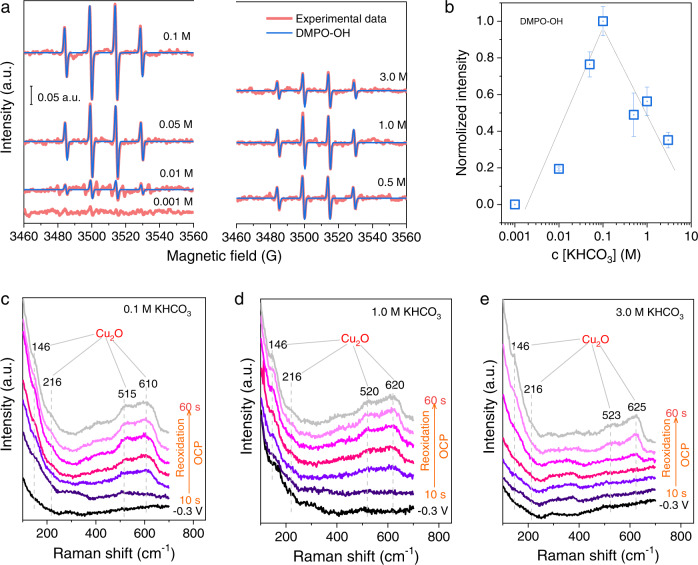
Relationships between HCO_3_^−^ concentration, OH^•^ radical amount, and reoxidation dynamics of OD-Cu electrodes. **a** The acquired EPR spectra in different HCO_3_^−^ concentrations. Each solution contains 100 mM DMPO. **b** Correspondingly, the normalized intensity of DMPO-OH versus HCO_3_^−^ concentration. The intensity was normalized by that of 0.1 M HCO_3_^−^. The gray lines guide the trends of DMPO-OH intensity without the compensation of K^+^. The error bars represent the standard deviation. **c**–**e** Raman spectra of OD-Cu reoxidation in different KHCO_3_ concentrations at OCP after reduction at −0.3 V_RHE_.

According to the above analysis, HCO_3_^−^ anions play a vital role in determining the OH^•^ generation. We thus studied the relationships between HCO_3_^−^ concentration, the intensity of OH^•^ radicals, and reoxidation dynamics on OD-Cu electrodes. Firstly, we investigated the influence of HCO_3_^−^ concentrations on the amount of OH^•^ radicals. As shown in Fig. 5a, at <0.1 M, the DMPO-OH signal increases with increasing the HCO_3_^−^ concentrations, yet further increasing the concentrations cannot produce more OH^•^ radicals. It is worth noting that further increasing the HCO_3_^−^ concentration enhances the K^+^ concentration as well. Thus, at the same K^+^ concentration (0.5 M) with K^+^ compensation by K_2_SO_4_, the relationship between the HCO_3_^−^ concentration and the intensity of DMPO-OH is more pronounced following the order 0.2 M < 0.4 M ≤ 0.5 M (Supplementary Fig. 10), and the intensity of DMPO-OH tends to saturate. The optimal HCO_3_^−^ concentration without K^+^ compensation for the OH^•^ radical formation is around 0.1 M (Fig. 5b).

Then, to correlate the OH^•^ radicals with reoxidation of OD-Cu electrodes, we carried out in situ Raman in different concentrations of HCO_3_^−^ solutions. As shown in Fig. 5c–e, with increasing the HCO_3_^−^ concentration from 0.1 to 3.0 M, the Raman intensity of Cu_2_O species decreases, and the reappearance of Cu_2_O phase delays, during the reoxidation processes after reduction at −0.3 V_RHE_. Thus, it is indeed that the intensity of DMPO-OH is very related to the reoxidation ability. These results suggest that HCO_3_^−^ contributes to the generation of OH^•^ radicals that oxidize the Cu surfaces.

**Fig. 6:**
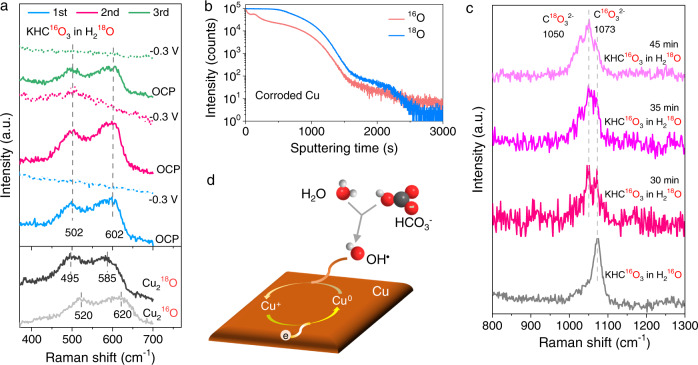
Determination of OH^•^ sources by oxygen isotope labeling in Ar-saturated 0.5 M KHC^16^O_3_ H_2_^18^O solution. **a** Raman spectra of surface Cu_2_O species under the loop tests with reduction at −0.3 V_RHE_ for 10 s and subsequently reoxidation at OCP for 20 s. The plots at the bottom show reference spectra for Cu_2_^16^O and Cu_2_^18^O. **b** ^16^O and ^18^O contents of the CuO_x_ species at the Cu plates after 24 h oxidizing corrosion, measured by TOF-SIMS. **c** Raman spectra of carbonate species on the Cu electrode. Each spectrum was obtained after resting the solution for the indicated time. The bottom spectrum shows C^16^O_3_^2−^ vibration as a reference. **d** The proposed dynamic reduction/reoxidation process of surface OD-Cu during CO_2_RR.

To clarify the sources of OH^•^ radicals, we applied a Raman measurement with oxygen isotope labeling in Ar-saturated 0.5 M KHC^16^O_3_ H_2_^18^O solution. The Raman vibrations of surface Cu_2_O species were recorded again by applying alternate potentials between −0.3 V_RHE_ and OCP. As shown in Fig. 6a, during the three cycles, the characteristic bands of Cu_2_O species at ~502 and ~602 cm^−1^ locate between those of Cu_2_^16^O (520, 620 cm^−1^) and Cu_2_^18^O (495, 585 cm^−1^). This indicates that both HC^16^O_3_^−^ anions and H_2_^18^O are responsible for the OH^•^ generation.

To further reveal the respective contributions of HC^16^O_3_^−^ and H_2_^18^O to OH^•^ radicals, we implemented time-of-flight secondary-ion mass spectrometry (TOF-SIMS) measurement to investigate the ^16^O and ^18^O contents of CuO_x_ species formed after 24 h oxidizing corrosion of Cu plate in Ar-saturated 0.5 M KHC^16^O_3_ H_2_^18^O solution. As illustrated in Fig. 6b, both ^16^O and ^18^O are detected in the whole sampling depth, and the latter has a little higher content. Concerning that pure water does not donate OH^•^ radicals (Fig. 4g and Supplementary Fig. 9), and the isotope oxygen mole ratio (HC^16^O_3_^−^ over H_2_^18^O is around 1:100), we hypothesize that oxygen exchange between H_2_^18^O and HC^16^O_3_^−^ may exist.

To support this hypothesis, we tracked the adsorbed carbonate species on the OD-Cu electrode in Ar-saturated KHC^16^O_3_ H_2_^18^O solution by using Raman spectroscopy. As displayed in Fig. 6c, within 30 min, both C^16^O_3_^2−^ vibration from pristine HC^16^O_3_^−^ at 1073 cm^−1^ and C^18^O_3_^2−^ vibration from ^18^O-derived HC^18^O_3_^−^ at 1050 cm^−1^ were detected^45^, indicating a fast oxygen exchange. After 45 min, dominant C^18^O_3_^2−^ vibration from ^18^O-exchanged HC^18^O_3_^−^ was observed. Thus, we show that the OH^•^ radicals have been generated at room temperature in HCO_3_^−^ electrolytes while the dynamic oxygen exchange between HCO_3_^−^ and H_2_O supplies oxygen sources for the formation of OH^•^ radicals.

Based on the above results, we proposed a mechanism for the dynamic reduction/reoxidation behavior at the OD-Cu surface during CO_2_RR in Fig. 6d. The reduction of Cu^δ+^ species to metallic Cu^0^ driven via cathodic reduction competes with the reoxidation of Cu^0^ to Cu^δ+^ state caused by the highly oxidative OH^•^ radicals. Thereby, there is a “seesaw-effect” between the reduction and reoxidation, determining the chemical state of Cu and the content of CuO_x_ species at the surfaces of Cu electrodes in CO_2_RR.

We discovered that strongly oxidative OH^•^ radicals can be easily generated in HCO_3_^−^ aqueous solutions at room temperature, and the fast oxygen exchange between HCO_3_^−^ and H_2_O provides dynamic oxygen sources for the OH^•^ radical formation. The generated OH^•^ radicals enable rapid reoxidation of OD-Cu electrodes in KHCO_3_ electrolytes during CO_2_RR. Besides, the continuous generation of OH^•^ radicals can make higher degrees of oxidizing corrosion of Cu electrodes in KHCO_3_ solutions to form substantial surface CuO_x_ species relative to those electrolytes without OH^•^ radicals. We further suggest that the dynamic chemical states of Cu and the content of surface CuO_x_ species are determined by a “seesaw-effect” between the cathodic reduction potentials and the OH^•^ radical-involving oxidation. This work provides insights into the reoxidation mechanism of OD-Cu and a general guide for understanding the crucial role of electrolyte composition for the CO_2_RR.

## Methods

### Electrodeposited Cu electrodes

All the electrochemical operations were performed using a Bio-logic SP200 potentiostat. A modified electrodeposition method was used to deposit micro-nano Cu particles at the Cu mesh substrate^29^. The copper mesh was ultrasonically cleaned in acetone, ethanol, and deionized water in sequence. After drying by flowing N_2_, the electrodeposition was carried out by applying a reduction current of −40 mA cm^−2^ for 20 min to the Cu mesh electrode in an Ar-saturated solution consisting of 0.1 M CuSO_4_·5H_2_O (>99.99%, Aladdin) and 1.5 M H_2_SO_4_ (>98%, Chron Chemicals). The as-prepared electrode was rinsed with water and ethanol sequentially and then dried under a stream of N_2_.

### Polished Cu plates

Cu plate with a thickness of ~0.2 mm was first polished by using 3000 mesh sandpaper and then cleaned in acetone, ethanol, and deionized water in sequence. Further, it was electropolished in 85% phosphoric acid (Chron Chemicals) solution at 3.0 V versus counter-electrode of another Cu plate for 5 min. After that, the polished Cu plate was ultrasonically cleaned in Ar-saturated ultrapure water (Milli-Q, 18.2 MΩ) to remove the surface residual particles.

### Oxidizing corrosion of polished Cu plates

The polished Cu plates were placed into CO_2_-, Ar-saturated 0.5 M KHCO_3_, CO_2_-saturated 0.5 M KHCO_3_ containing 10 mM VC, Ar-saturated ultrapure water, Ar-saturated 0.25 M K_2_SO_4_, and Ar-saturated 0.5 M KHCO_3_ H_2_^18^O solutions, respectively. After resting for 24 h, the corrosive Cu plates were rinsed with water and then dried under a stream of N_2_.

### Materials characterization

Crystal phase structures were characterized by an XRD diffractometer (Equinox 1000, Thermo Fisher Scientific) with Cu Kα radiation (*λ* = 1.54 Å). Morphology was observed by field-emission SEM (FEI Inspect F50). Elemental analysis was implemented by using EDS. TEM and HRTEM images were acquired by using an FEI Titan G2 60-300 electron microscope. The surface chemistry of the Cu electrode/plate was investigated using XPS (Thermo ESCALAB 250XI) with Al Kα X-rays (1486.6 eV). The depth ^16^O and ^18^O contents of CuO_x_ species formed at the surface of the Cu plate were analyzed by applying TOF-SIMS (ION TOF-SIMS 5) with 30 keV-Bi_3_^+^ as an analysis gun and 2 keV-Cs^+^ as the sputtering source.

### In situ Raman spectroscopy

Raman spectra were recorded with an XploRA PLUS Raman spectrometer (Horiba Jobin Yvon) equipped with a ×50 objective and a 638 nm He-Ne laser. The filter was set at 50%. The measurements were conducted using a custom-made three-electrode electrochemical cell with a quartz window, in which the as-prepared Cu electrode, Ag/AgCl (3.0 M KCl), and membrane-separated Pt wire were used as the working, reference, and counter electrodes. Before each test, the as-used electrolyte was pumped into the cell at a rate of 2 ml min^−1^. The equipped optical microscope was applied to acquire the real-time microscopic images of as-used Cu electrodes during the Raman tests.

### EPR spectroscopy

EPR measurements were carried out using a continuous-wave Bruker EMX micro spectrometer operating in X-band mode with a frequency of 9.848 GHz at room temperature. Each spectrum was recorded using the following parameters: a microwave power of 20 mW, modulation amplitude of 1.0 G, and a single scan with a sweep time of 5 min. DMPO (Dojindo) was selected as the spin trapping agent, and its concentration in all solutions was 100 mM. For EPR measurements taken during electrocatalysis in the presence of a Cu electrode, the electrolyte was immediately measured after electrolysis at a given potential in a specific electrolyte containing 100 mM DMPO. EPR simulations were performed according to the hyperfine splitting constants of radicals, via using the Xenon software provided by the EPR manufacturer.

## Supplementary information

Supplementary information

Peer Review File

## Data Availability

All the data that support the findings of this study are available within the paper and its Supplementary Information files, or from the corresponding author on reasonable request.
