# Supplementary material for: Hydroxyl radicals dominate reoxidation of oxide-derived Cu in electrochemical CO2 reduction
Source: Nat Commun. 2022 Jun 27;13:3694. doi: 10.1038/s41467-022-31498-8 (PMC9237086; doi:10.1038/s41467-022-31498-8)
Supplement: Supplementary file 1 — Supplementary information [file 41467_2022_31498_MOESM1_ESM.pdf]

Supporting Information

Hydroxyl radicals dominate reoxidation of oxide-derived Cu in electrochemical CO_2 reduction

Shijia Mu[1], Honglei Lu[1], Qianbao Wu[1], Lei Li[1], Ruijuan Zhao[1], Chang Long[1,*], Chunhua Cui[1,*]

[1]Molecular Electrochemistry Laboratory, Institute of Fundamental and Frontier Sciences, University of Electronic Science and Technology of China, Chengdu 610054, China.

*Corresponding authors:
Chang Long – *E-mail: longch@uestc.edu.cn*
Chunhua Cui – *E-mail: chunhua.cui@uestc.edu.cn*

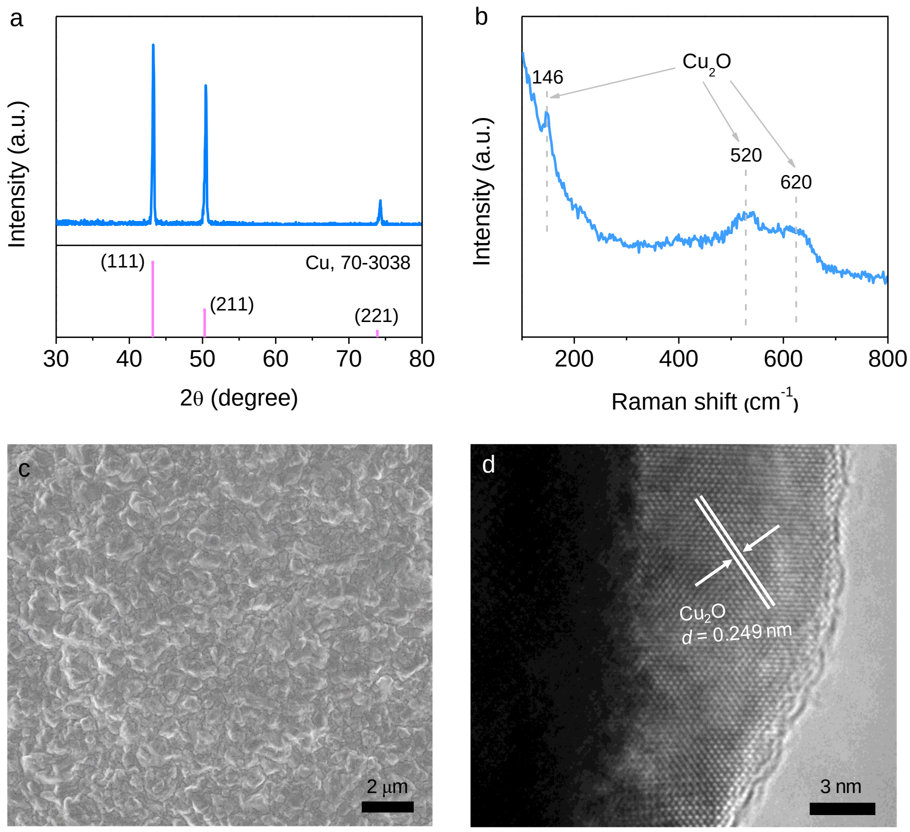

Supplementary Fig. 1. Characterizations of the as-prepared OD-Cu electrodes. (**a**) XRD pattern, (**b**) Raman spectrum, (**c**) SEM and (**d**) HRTEM images.

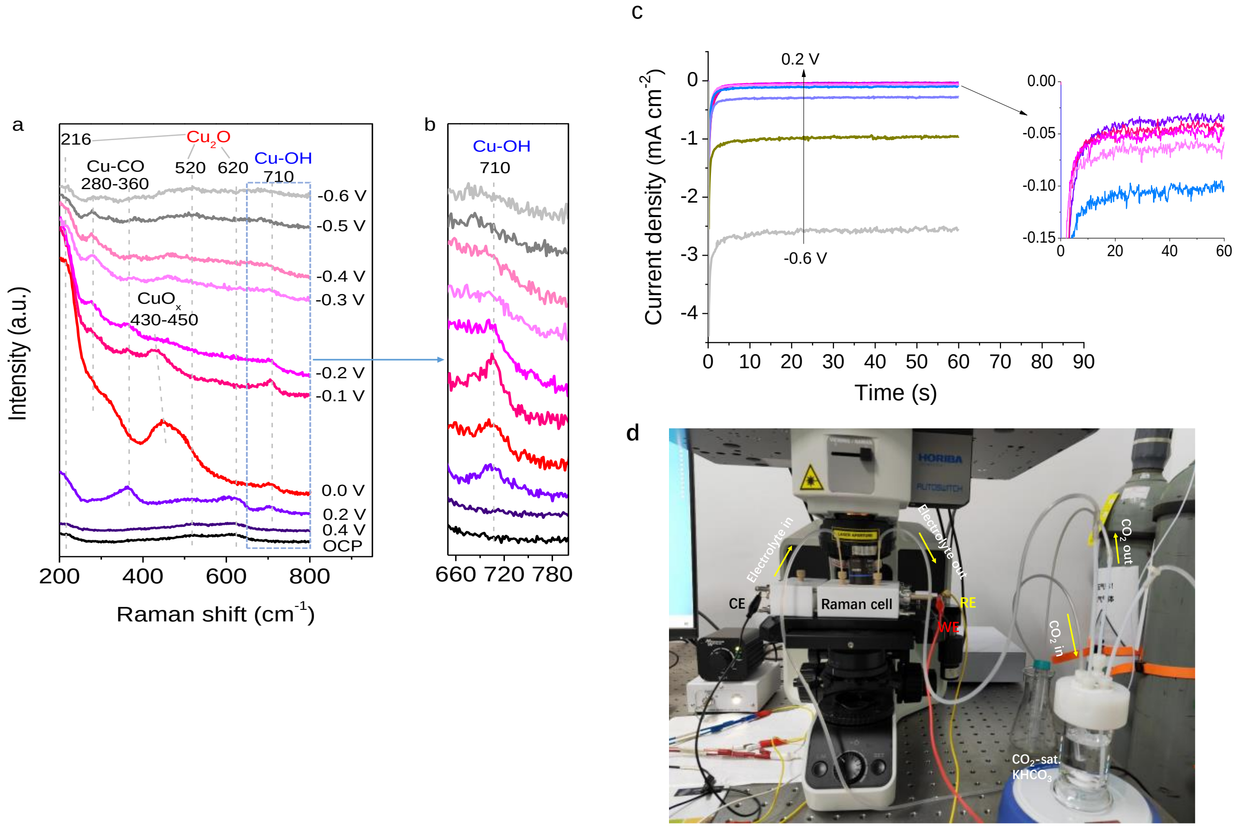

Supplementary Fig. 2. Raman spectra of surface species at OD-Cu electrode under electrolysis at indicated potentials versus RHE in a CO_2-saturated 0.5 M $KHCO_3$ solution. (**a**) Vibration bands of the surface Cu_2O, generated intermediate CO, and surface hydroxyl species that was assigned as Cu-OH modes. (**b**) The zoom-in view of Cu-OH was marked by a dashed box in (**a**). (**c**) Current-time curves at the indicated potentials versus RHE for potential-dependent Raman tests in CO_2-saturated 0.5 M $KHCO_3$ solution. Inset shows a zoom-in view. (**d**) Experimental setup image of *in-situ* electrochemical Raman spectroscopy.

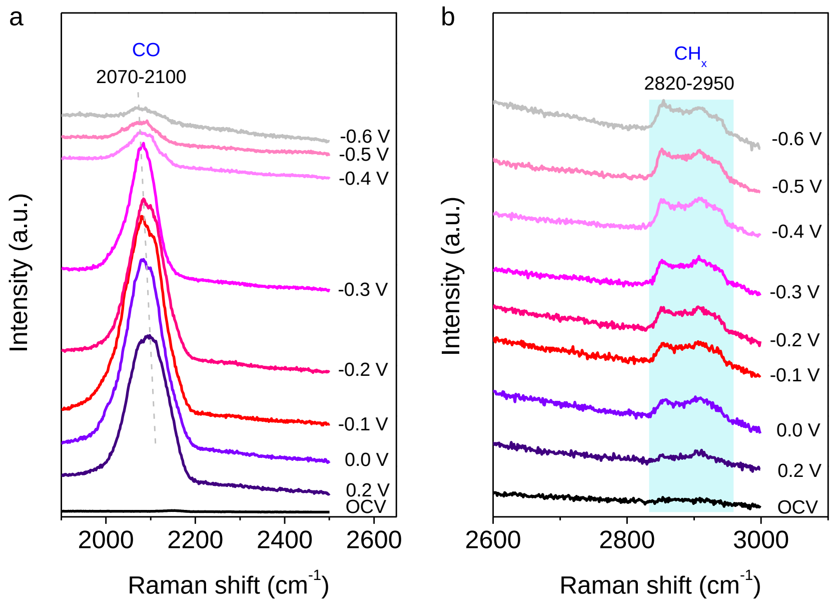

Supplementary Fig. 3. Raman spectra of the generated CO_2RR intermediates at OD-Cu electrode surface under electrolysis at indicated potentials versus RHE in the CO_2-saturated 0.5 M $KHCO_3$ solution. Vibration bands of the (**a**) intramolecular C≡O intermediate and (**b**) CH_x intermediates.

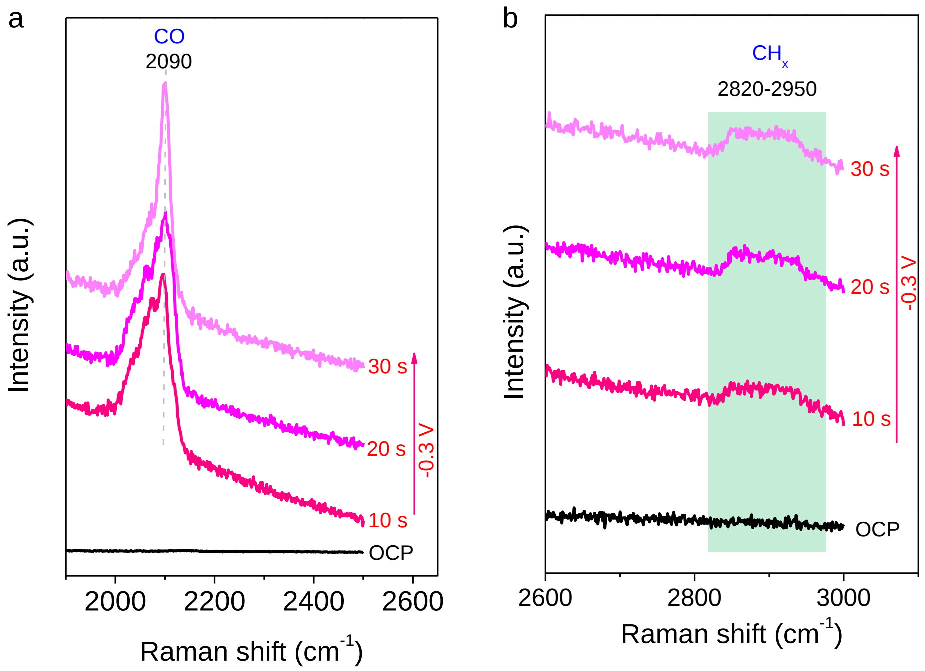

Supplementary Fig. 4. *In-situ* Raman spectra of the generated CO_2RR intermediates at OD-Cu electrode surface under electrolysis at -0.3 V versus RHE for different periods in the CO_2-saturated 0.5 M $KHCO_3$ solution. Vibration bands of the (**a**) intramolecular C≡O intermediate and (**b**) CH_x intermediates.

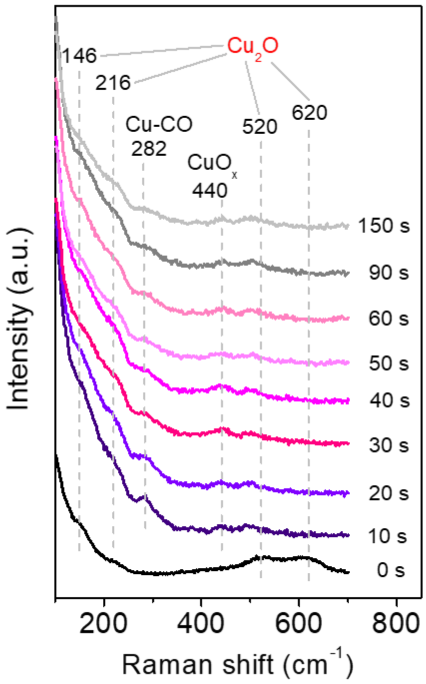

Supplementary Fig. 5. Time-dependent Raman spectra of surface Cu_2O species at -0.3 V_{RHE} in the CO_2-saturated 0.5 M $KHCO_3$ solution.

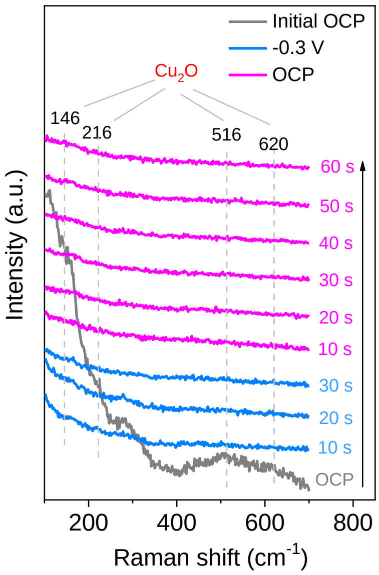

Supplementary Fig. 6. Real-time Raman spectra of surface Cu_2O species at -0.3 V_{RHE} and subsequently at OCP in the Ar-saturated 0.25 M K_2SO_4 solution.

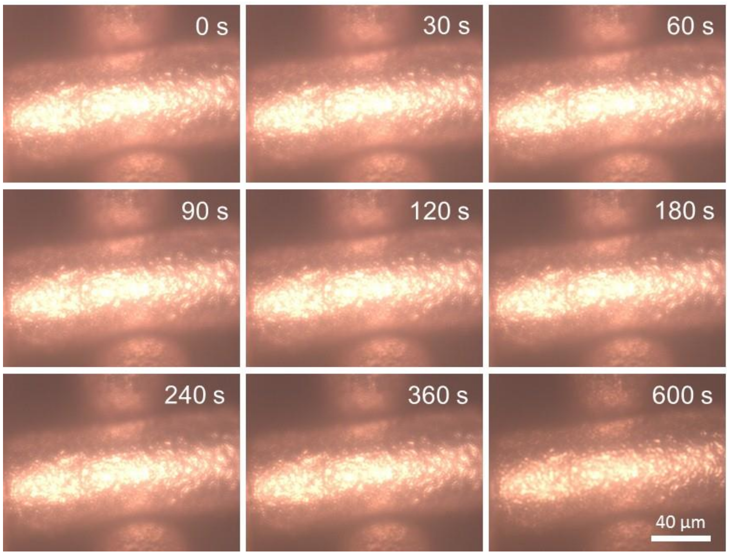

Supplementary Fig. 7. Time-dependent optical-microscopic images of OD-Cu electrodes after switching the potential from -0.3 V_{RHE} (20 min) to OCP.

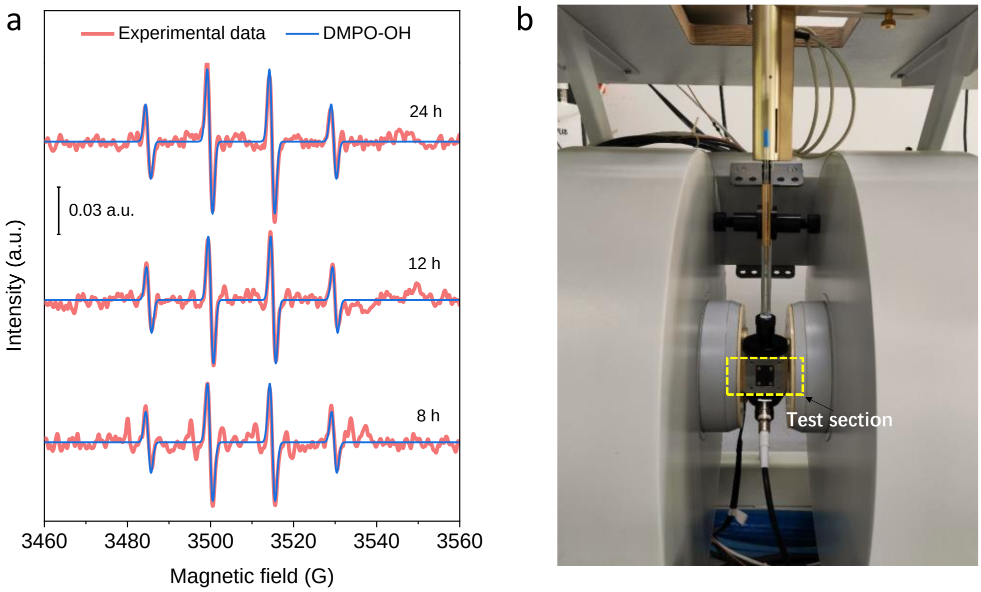

Supplementary Fig. 8. (a) Time-dependent EPR spectra of the CO_2-saturated 0.5 M $KHCO_3$ solution containing 100 mM DMPO at OCP. **(b)** Image of EPR instrument equipped with the capillary tube upon measurement.

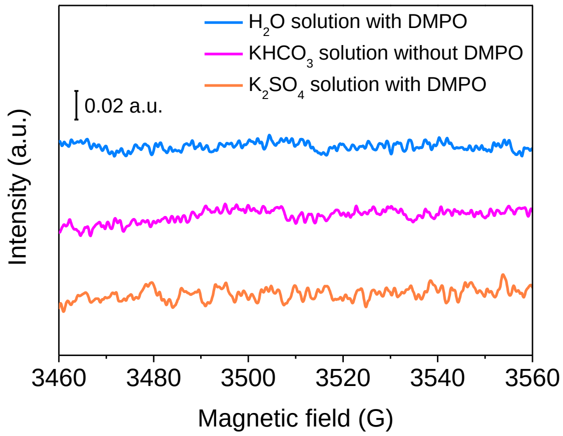

Supplementary Fig. 9. EPR spectra of the water solution, 0.5 M $KHCO_3$ solution, and 0.25 M K_2SO_4 solution containing 100 mM DMPO.

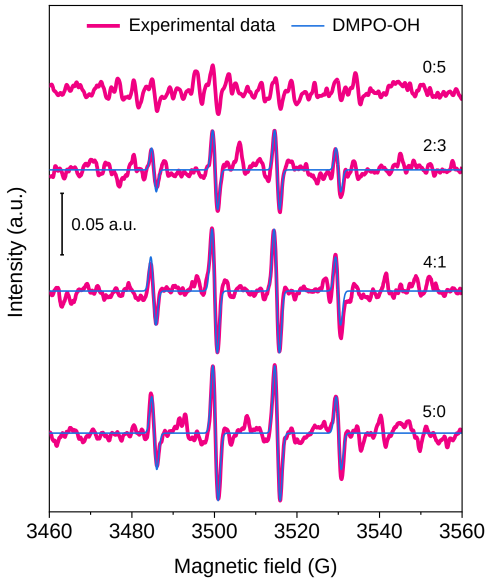

Supplementary Fig. 10. EPR spectra of the $KHCO_3/K_2SO_4$ mixed solutions after 24 resting at OCP. The solutions at different HCO_3^-/SO_4^{2-} mole ratios contain 100 mM DMPO under the same K^+ concentrations (0.5 M).

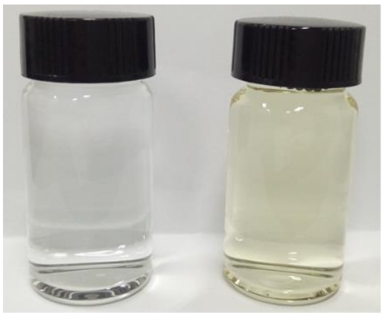

Supplementary Fig. 11. Photographs of CO_2-saturated 0.5 M $KHCO_3$ solution containing 10 mM VC. Before (left) and after (right) 24 h aging.

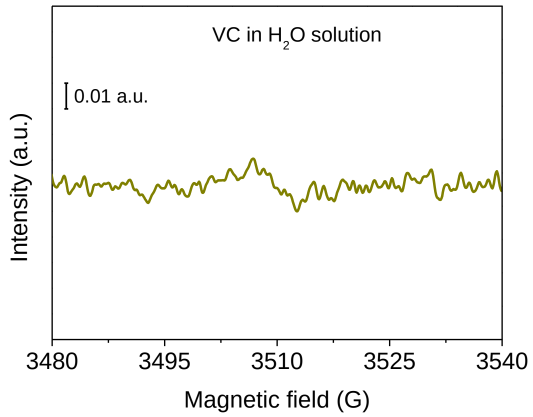

Supplementary Fig. 12. EPR spectra of the 10 mM VC water solution containing 100 mM DMPO.

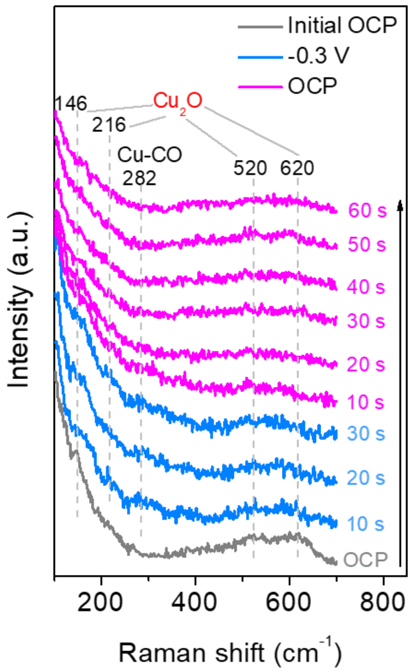

Supplementary Fig. 13. Real-time Raman spectra of surface Cu_2O species at -0.3 V_{RHE} and subsequently at OCP in CO_2-saturated 0.5 M $KHCO_3$ solution containing 10 mM VC.

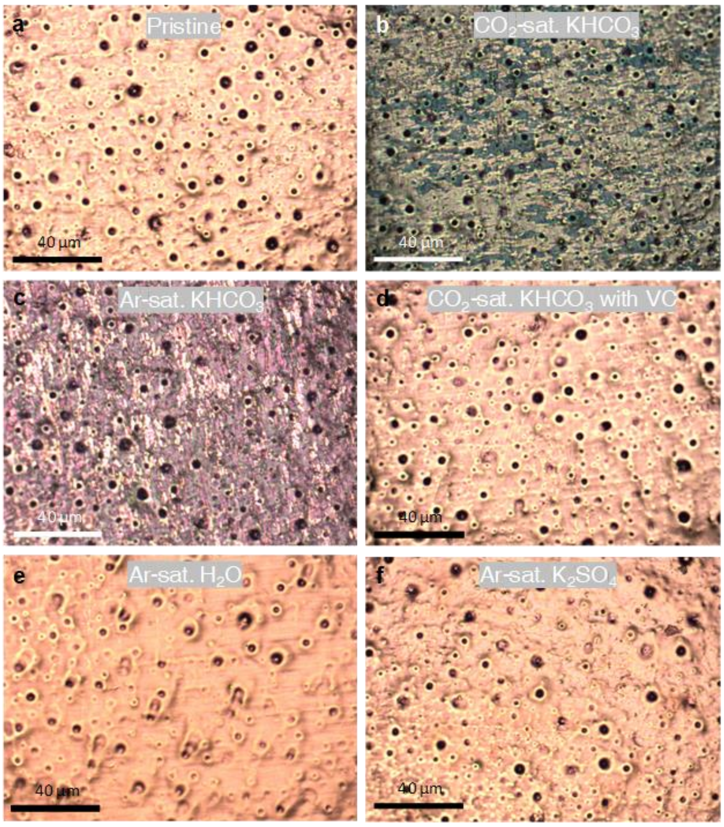

Supplementary Fig. 14. Optical microscopic images of the polished Cu plates. Before (**a**) and after 24 h oxidizing corrosions in (**b**) CO_2-saturated $KHCO_3$, (**c**) Ar-saturated $KHCO_3$, (**d**) CO_2-saturated $KHCO_3$ containing VC, (**e**) Ar-saturated pure water, and (**f**) Ar-saturated K_2SO_4 solutions. The concentrations of $KHCO_3$, K_2SO_4, and VC are 0.5 M, 0.25 M, and 10 mM, respectively.

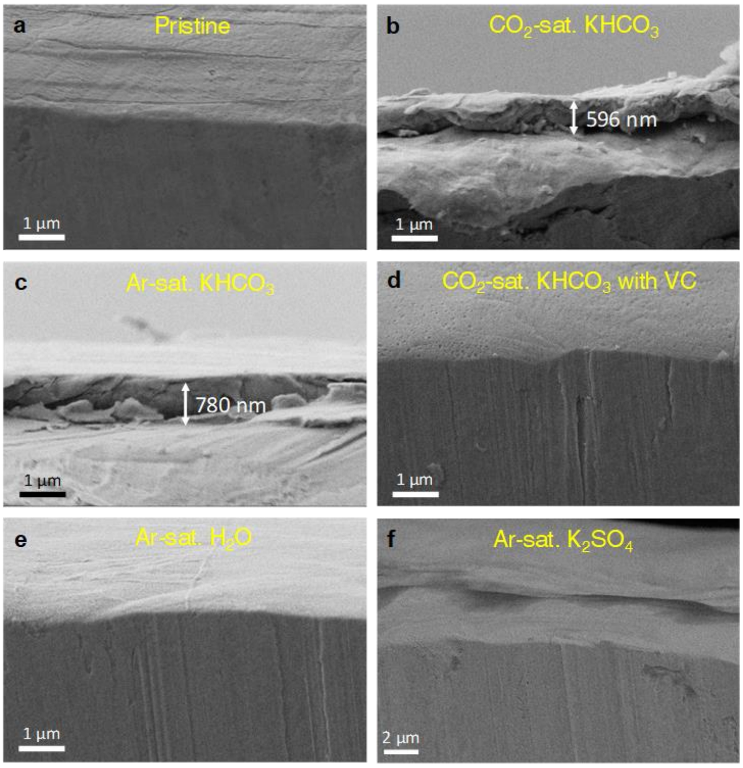

Supplementary Fig. 15. SEM images of the cross-sections of polished Cu plates. Before (**a**) and after 24 h oxidizing corrosions in (**b**) CO_2-saturated $KHCO_3$, (**c**) Ar-saturated $KHCO_3$, (**d**) CO_2-saturated $KHCO_3$ containing VC, (**e**) Ar-saturated pure water, and (**f**) Ar-saturated K_2SO_4 solutions. The concentrations of $KHCO_3$, K_2SO_4, and VC are 0.5 M, 0.25 M, and 10 mM, respectively.

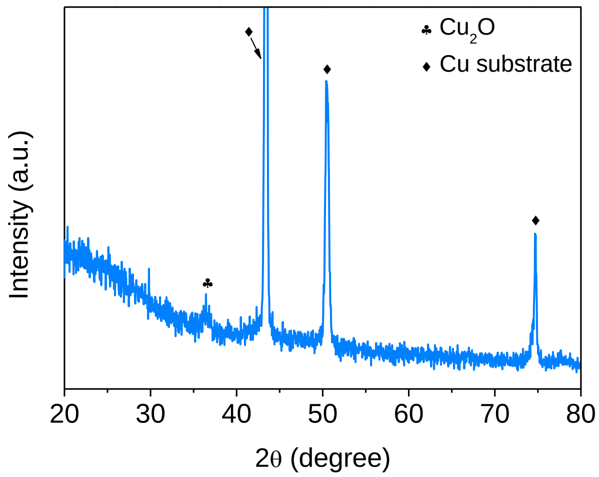

Supplementary Fig. 16. XRD pattern of Cu plate after 24 h corrosion in 0.5 M CO_2-saturated $KHCO_3$ solution.

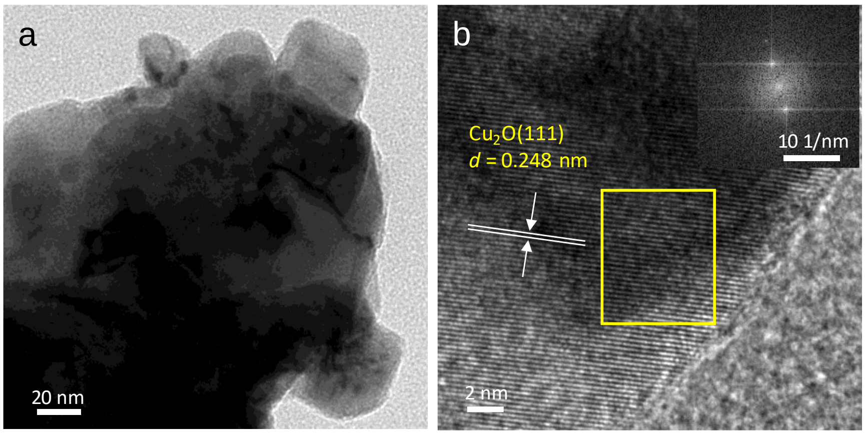

Supplementary Fig. 17. Characterizations of the corrosion products at Cu plate surface after resting in CO_2-saturated $KHCO_3$ solution for 24 h. (**a**) TEM and (**b**) HRTEM images. Inset in (**b**) is the fast Fourier transforms of the images marked by the rectangle.

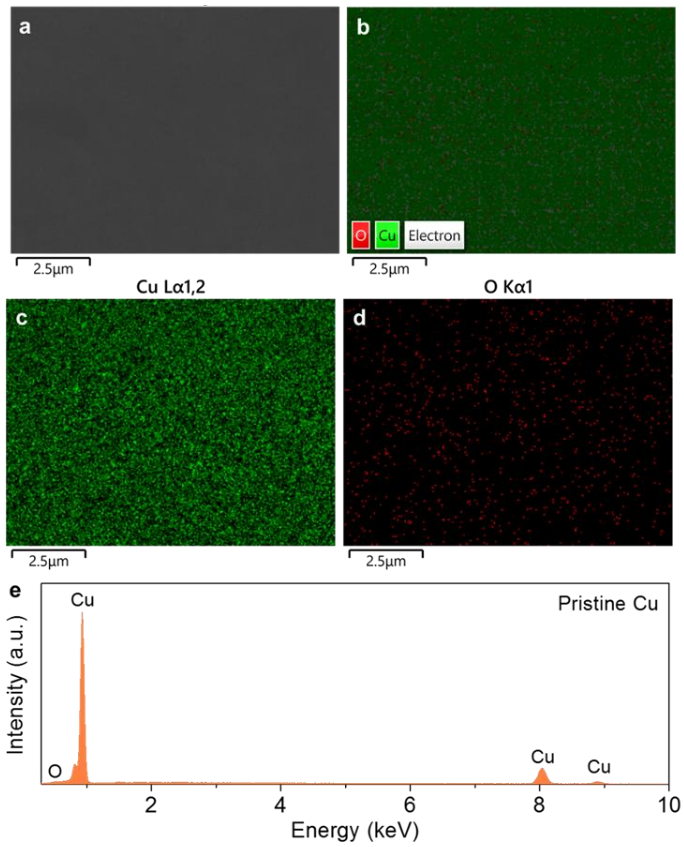

Supplementary Fig. 18. EDS of the pristine polished Cu plate. (**a-d**) mapping and (**e**) spectrum.

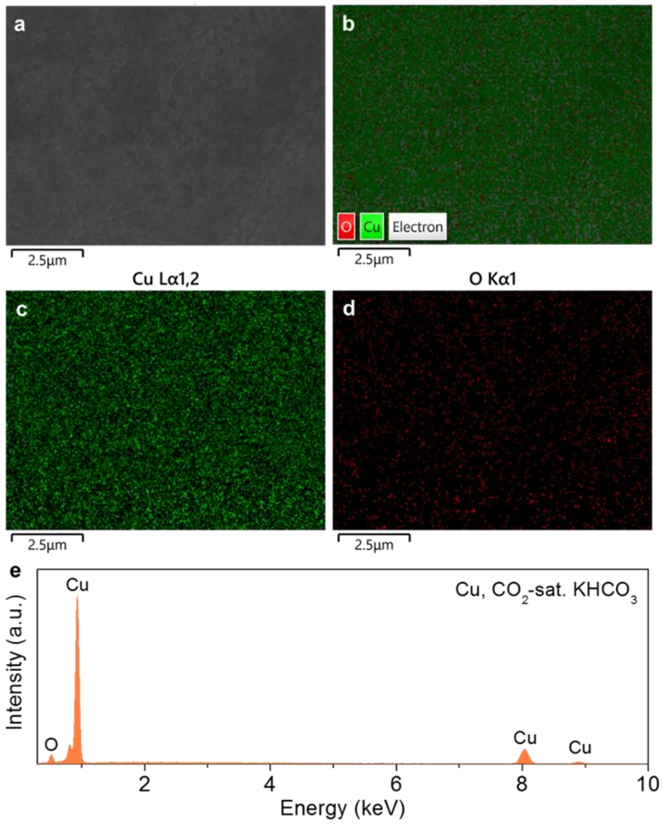

Supplementary Fig. 19. EDS of the Cu plate after 24 h oxidizing corrosion in CO_2-saturated 0.5 M $KHCO_3$ solution. (**a-d**) mapping and (**e**) spectrum.

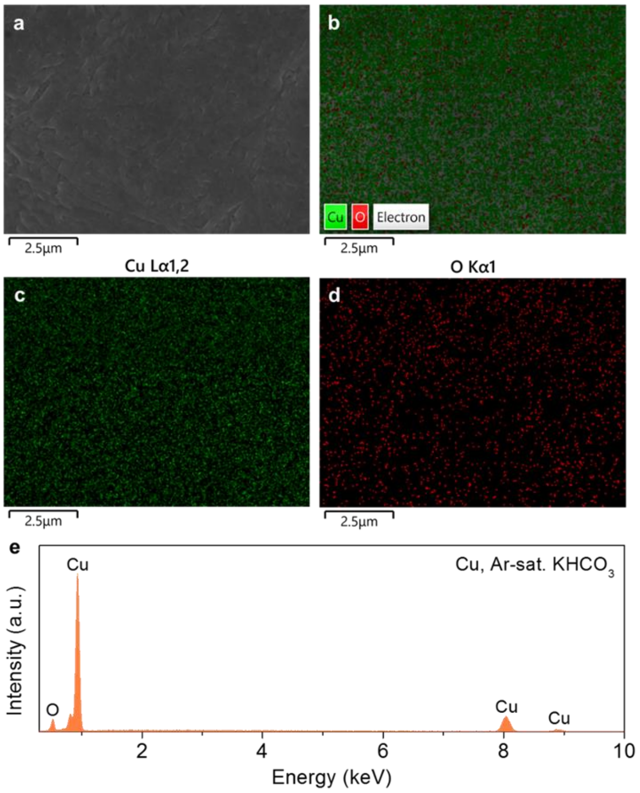

Supplementary Fig. 20. EDS of the Cu plate after 24 h oxidizing corrosion in Ar-saturated 0.5 M $KHCO_3$ solution. (**a-d**) mapping and (**e**) spectrum.

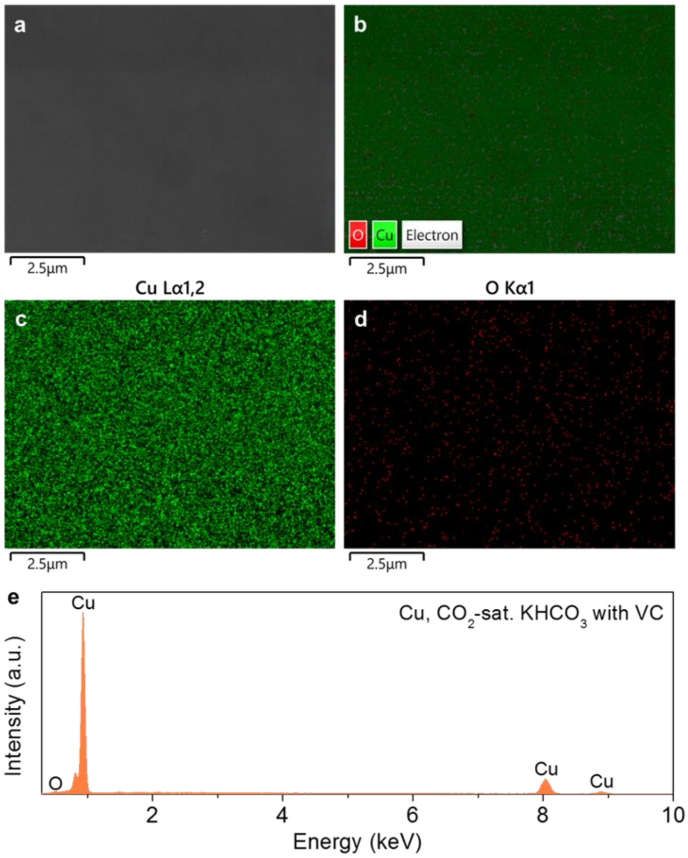

Supplementary Fig. 21. EDS of the Cu plate after 24 h oxidizing corrosion in CO_2-saturated 0.5 M $KHCO_3$ solution containing 10 mM VC. (**a-d**) mapping and (**e**) spectrum.

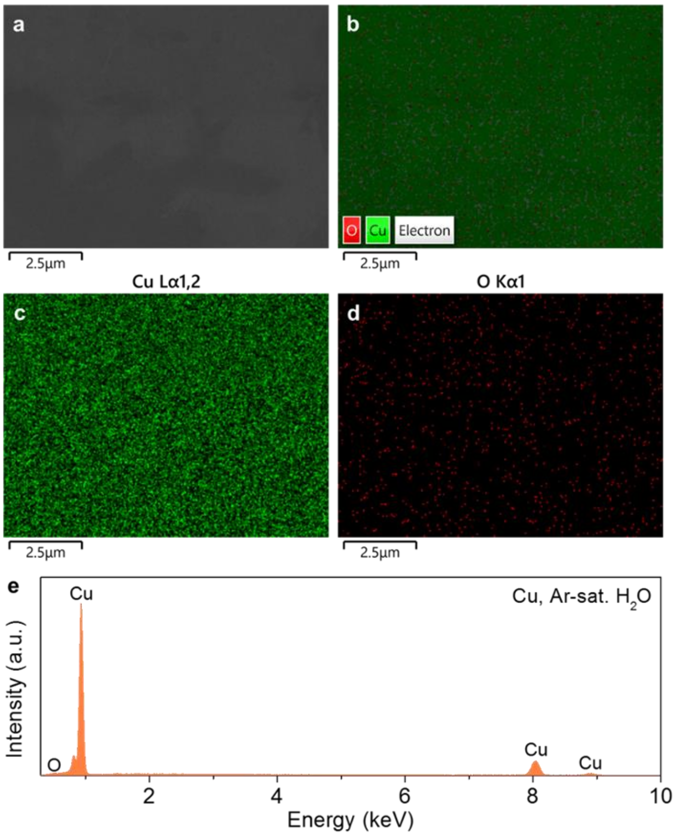

Supplementary Fig. 22. EDS of the Cu plate after 24 h oxidizing corrosion in Ar-saturated pure water solution. (**a-d**) mapping and (**e**) spectrum.

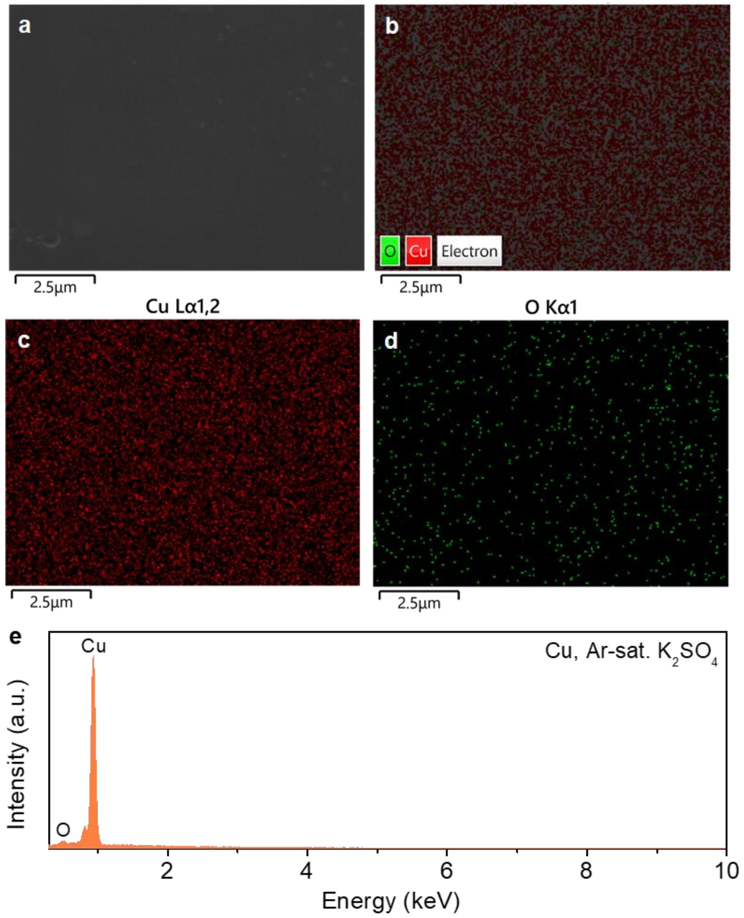

Supplementary Fig. 23. EDS of the Cu plate after 24 h oxidizing corrosion in Ar-saturated 0.25 M K_2SO_4 solution. (a-d) mapping and (**e**) spectrum.

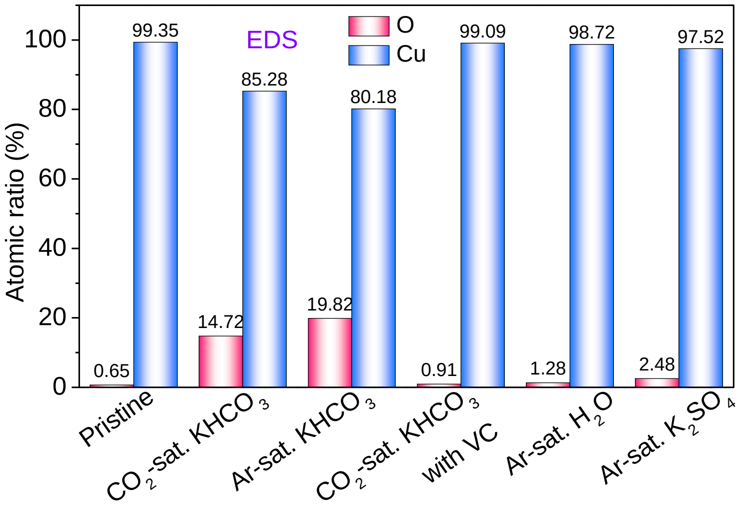

Supplementary Fig. 24. Atomic contents of Cu and O elements are determined by EDS.

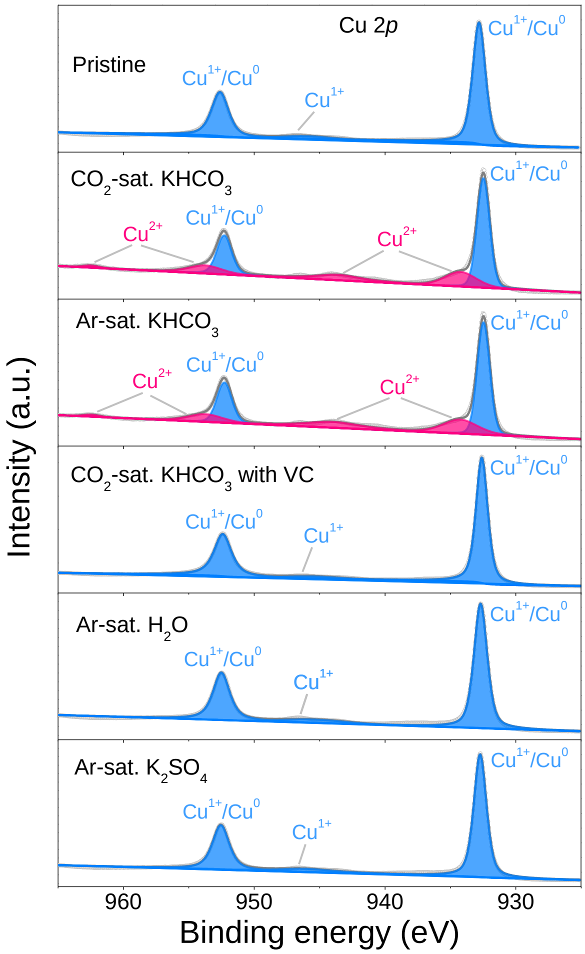

Supplementary Fig. 25. XPS Cu 2*p* spectra of Cu plates. Before (pristine), and after 24 h oxidizing corrosions in CO_2-saturated $KHCO_3$, Ar-saturated $KHCO_3$, CO_2-saturated $KHCO_3$ containing VC, Ar-saturated pure water, and Ar-saturated K_2SO_4 solutions. The concentrations of $KHCO_3$, K_2SO_4, and VC are 0.5 M, 0.25 M, and 10 mM, respectively.
